# Establishment of online deep learning model for insect-affected pests in “Yali” pears based on visible-near-infrared spectroscopy

**DOI:** 10.3389/fnut.2022.1026730

**Published:** 2022-10-24

**Authors:** Yong Hao, Chengxiang Zhang, Xiyan Li, Zuxiang Lei

**Affiliations:** ^1^School of Mechatronics and Vehicle Engineering, East China Jiaotong University, Nanchang, China; ^2^Key Laboratory of Conveyance Equipment of the Ministry of Education, Nanchang, China; ^3^School of Civil Engineering and Architecture, East China Jiaotong University, Nanchang, China

**Keywords:** insect-affected pears, Vis-NIR spectroscopy, CBAM attention module, online discrimination model, deep learning model

## Abstract

Insect-affected pests, as an important indicator in inspection and quarantine, must be inspected in the imports and exports of fruits like “Yali” pears (a kind of duck head-shaped pear). Therefore, the insect-affected pests in Yali pears should be previously detected in an online, real-time, and accurate manner during the commercial sorting process, thus improving the import and export trade competitiveness of Yali pears. This paper intends to establish a model of online and real-time discrimination for recessive insect-affected pests in Yali pears during commercial sorting. The visible-near-infrared (Vis-NIR) spectra of Yali samples were pretreated to reduce noise interference and improve the spectral signal-to-noise ratio (SNR). The Competitive Adaptive Reweighted Sampling (CARS) method was adopted for the selection of feature modeling variables, while Partial Least Squares Discriminant Analysis (PLS-DA), Support Vector Machine (SVM), and Convolutional Block Attention Module-Convolutional Neural Networks (CBAM-CNN) were used to establish online discriminant models. T-distributed Stochastic Neighbor Embedding (T-SNE) and Gradient-weighted Class Activation Mapping (Grad-CAM) were used for the clustering and attention distribution display of spectral features of deep learning models. The results show that the online discriminant model obtained by SGS pretreatment combined with the CBAM-CNN deep learning method exhibits the best performance, with 96.88 and 92.71% accuracy on the calibration set and validation set, respectively. The prediction time of a single pear is 0.032 s, which meets the online sorting requirements.

## Introduction

Yali pears (duck head-shaped pears) are rich in nutrients, tasting good, and have a huge consumer market (1). However, because of careless nursing, pear trees are subject to damages caused by insect pests such as pirivorella, grapholita molesta busck, and halyomorpha picus. These insect pests are widely distributed in temperate and subtropical regions of Asia, Europe, America, Africa, and Australia (2). In “the hometown of Yali pears,” Hebei province in China, insect-affected pests in Yali pears are so rampant that the damage rate can reach more than 85% due to improper management, which seriously undermines the quality of Yali pears, resulting in shrinking sales (3). The quality classification of Yali pears mainly includes two indicators, internal quality and external quality. The external quality of Yali pears mainly adopts manual and mechanical classification. Manual classification has many shortcomings such as high labor cost, low efficiency, unclear classification standard and lack of objectivity. As the production volume increases, the traditional manual classification can no longer satisfy the production demands (4). In contrast, classification by machines has higher efficiency, and the size, surface quality and weight of Yali pears are usually analyzed with the help of sensors such as image and gravity. The internal quality of Yali pears is usually detected by non-destructive and destructive methods. The destructive methods are only suitable for limited scenarios such as sampling or initial screening. But the non-destructive methods, if combined with sophisticated mechanical structure, can realize full-coverage detection of the samples. However, the key to successful application of non-destructive methods lies in the correct analysis of sensing signals by machine learning method (5). In recent years, the international community has carried out strict regulations on the defects of exported Yali pears. According to Chinese regulations on quarantine techniques of exported Yali pears, Yali pears cannot be sampled for quarantine inspection unless they have been stored in refrigerator for at least 21 days. Each batch of pears is sampled at 2% of the total number of all boxes, and all samples are detected. Lengthy inspection and quarantine process leads to higher export costs and deterioration risks. For this reason, there is an urgent need to figure out a rapid and non-destructive mechanical sorting method for full-coverage inspection of recessive defects in Yali pears, so as to improve the export quality and qualified rate of Yali pears.

Visible-near-infrared (Vis-NIR) spectroscopy is a non-destructive detection method which has been widely used in the internal quality detection of fruits and crops (6–8). Compared with other non-destructive detection methods such as machine vision (9), X-ray (10), and magnetic resonance imaging (MRI) (11), Vis-NIR spectroscopy has higher speed, lower costs and wider application of detection. According to the literature research, there are few studies on Vis-NIR detection of recessive pests in fruits and crops. Khodabakhshian et al. (12) detected Carob moths in pomegranates, and they found that the identification rate of insect pests in the combined sample sets through PLS-DA method could reach as high as 86%. Abbaspour-Gilandeh et al. (13) detected codling moths in red apples by digital image processing and sparse coding method, and the pest identification rates in healthy apples and diseased apples reached 81 and 86%. Moscetti et al. (14) detected Tortrices and Weevil in chestnuts, and the total error of identification was reduced to 8.41% by the Genetic Algorithm - Linear Discriminant Analysis (GA-LDA) method. The above researchers have proved that it is feasible to detect recessive insect pests in fruits by using Vis-NIR spectroscopy. Be that as it may, few researches have been conducted on Vis-NIR online identification of recessive insect pests inside Yali pears.

In this paper, an online rapid non-destructive detection method for internal defects of Yali pears based on Vis-NIR has been proposed, in a bid to tackle the online rapid screening of recessive Yali pear pests in the commercial sorting process. First, different pretreatment methods are used to suppress noise and enhance information on the original spectrum to improve the signal-to-noise ratio of the spectrum, such as Savitzky-Golay Smoothing (SGS), Spectral Standardization (SS), Max-min Normalization (MMN) and Standard Normal Variate Transformation (SNV). Second, Competitive Adaptive Reweighted Sampling (CARS) was used for the optimal selection of feature variables. Last, Partial Least Squares Discriminant Analysis (PLS-DA) and Support Vector Machines (SVM) were used to establish shallow learning model of online discrimination for Yali pears, and Convolutional Block Attention Module-Convolutional Neural Networks (CBAM-CNN) was used to establish deep learning model for online discrimination of Yali pears. This paper intends to figure out a method to establish an accurate and rapid online discrimination model for recessive insect pests inside fruits, in a bid to improve the quality sorting of fresh fruits.

## Samples and methods

### Spectral acquisition device

**FIGURE 1:**
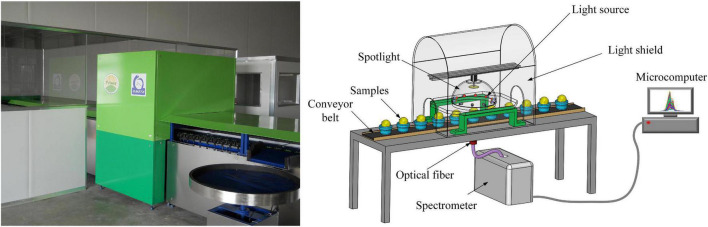
Schematic diagram of the Vis-NIR online sorting device for Yali pears.

**FIGURE 2:**
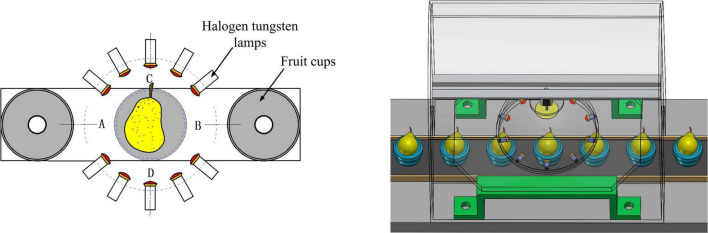
Schematic diagram of light source (halogen tungsten lamps) layout.

The online sorting device for insect-affected Yali pears is shown in Figure 1. The device is composed of five parts: spectrometer, optical fiber, microcomputer, light source and conveyor belt. The spectrometer is QE6500 Pro produced by the Ocean optics INC; the detector range is 200-1100 nm; the integration time of the acquisition device is set to 80 ms; the conveyor transports six samples per second; the optical fiber probe is located below the fruit cup, 12 mm from the conveyor belt. The light source consisted of ten 100-Watt halogen tungsten lamps (Osram) positioned above the sample and arranged along the concentrating coil with an oblique angle of 45 degrees. The light sources are arranged as shown in Figure 2, with five light sources equally spaced on each side around the central spectral acquisition station.

Prior to spectral acquisition, the parameters of the spectrometer must be calibrated. First, the light sources were turned on for 30 min to ensure the thermal stability of light sources and detector. Second, a PTFE ball with a diameter of about 70 mm was used to calibrate each fruit cup, so as to maintain the consistency of measurement background. In the fruit cup, the pear samples were placed in a way that the connection direction between the pear stalk (C) and the pear pedicel (D) was always perpendicular to the running direction of the conveyor belt.

The working process of the Vis-NIR online sorting device for Yali pears is shown as follows. The Yali pear samples are placed according to Figure 2. In other words, the connection direction between the pear stalk (C) and the pear pedicel (D) was always perpendicular to the running direction of the conveyor belt. When the sample reaches the spectral acquisition position, the light source penetrates the inside of the pear and then reaches the optical detector. The whole spectral acquisition process is completed in the light shield, which effectively reduces the disturbances of external stray light to spectral signals.

### Preparation of Yali pear samples and discrimination of insect pests

**FIGURE 3:**
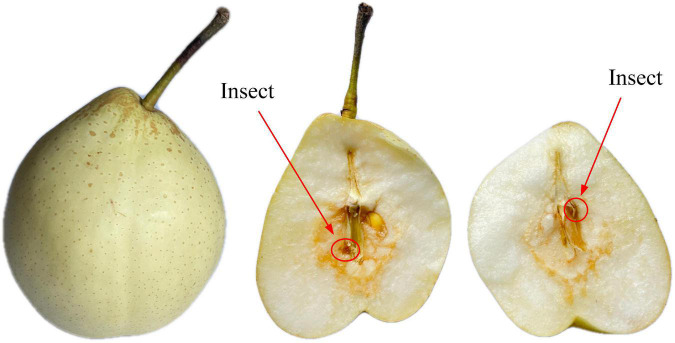
Insect-affected Yali pear samples.

Samples for experiment are Yali pears produced in Hebei province, with a total number of 960. These samples were transported in refrigerated vehicles to laboratory, and then were stored at a constant temperature of 20° for 21 days. Prior to experiment, the stains and moisture on the surface of Yali pear samples were removed. After collecting the samples of the Vis-NIR spectrum, the artificial incision identification method was used to identify the internal pests of the pear. When cutting Yali pears, the first cutting was conducted along the A–B connection direction, and the second cutting was conducted along the C–D connection direction, as shown in Figure 2. Observing carefully whether there were indicators of insect decay inside the pear and insect holes on the surface. Evaluated whether it was insect-affected pears or not and decided by three experts with years of experience in planting and sales of Yali pears. Some of the insect-affected Yali pear samples are shown in Figure 3 (The area indicated by the red circle).

### Classification of sample sets

A total of 960 Yali pear samples were divided at a ratio of 4:1 into calibration set and validation set using SPXY algorithm. SPXY algorithm is developed on the basis of KS algorithm, and its principle is to ensure the difference and representativeness of samples by calculating the distance between the spectral feature values of different samples. SPXY can effectively cover the multi-dimensional vector space to avoid the over-fitting or poor prediction effect of the prediction model caused by too small or identical differences between samples, so as to improve the stability and accuracy of the model (15). The information of sample sets is shown in Table 1, the calibration set includes 768 samples (468 healthy pears and 300 insect-affected pears); the validation set includes 192 samples (124 healthy pears and 68 insect-affected pears).

**TABLE 1:** Sample set information.

Sample set	Healthy pears	Insect-affected pears	Total number	Proportion/%
Calibration set	468	300	768	80%
Validation set	124	68	192	20%

### Methods of spectral pretreatment and feature variable selection

As shown in Figure 1, the online detection device is an open system and the Yali pear itself is not an ellipsoid with a regular shape. Therefore, in the process of spectral signal acquisition, it is often disturbed by stray light, noises, baseline drift and so on, thus affecting the final quantitative and qualitative analysis results. For this reason, it is necessary to pretreat original spectra and optimally select feature variables before modeling, so as to eliminate or reduce the interference of invalid information to pear spectra (16). Taking into account the surface morphology of pear samples and the structure of spectral acquisition device, SGS smoothing was used to eliminate the random noises in spectral signals and improve the SNR of samples (17). Spectral Standard (SS) was adopted to reduce the scale difference in spectral signals (18). Max-min Normalization (MMN) was adopted to eliminate the baseline drift and irrelevant noise signals with interference in spectral signals (19). The scattering effect caused by uneven particle distribution and different particle size in the spectral signal was eliminated through the Standard Normal Variate (SNV) transformation (20).

For the feature variable selection method, CARS method is one of the most commonly used methods in the selection of fruit spectral variables, which combines Monte Carlo sampling and PLS model regression coefficients (21). Compared with other variable selection methods like Principle Component Analysis (PCA), Uninformative Variable Elimination (UVE), and Successive Projections Algorithm (SPA), the CARS method is more convenient and the feature variables selected by the CARS are more representative, which can effectively reduce the high collinearity between wavebands caused by the number of spectral wavelength points greater than the number of samples. In this way, the prediction accuracy and speed of model can be improved (22).

### Establishment of discrimination model for insect-affected Yali pears

#### Shallow learning method

Partial Least Squares Discriminant Analysis (PLS-DA) is a multivariate statistical method under supervision, which integrates the basic functions of PCA, canonical correlation analysis and multiple regression analysis, and can compress data and extract feature information (23). The principle of PLS-DA is to separately train the features of samples processed by different methods, so as to generate the calibration set and test its confidence. This method can group the required observation variables in advance, and conduct statistical analysis on the data according to the nature of the grouping, so as to learn the key variables that affect the grouping (24). PLS-DA is usually used for classification and discrimination. When the difference between groups is small and the sample size of each group varies significantly, this method can effectively distinguish the observed variables between groups, and its linear classification feature is widely used in Vis-NIR qualitative analysis.

Support Vector Machine (SVM) is an algorithm based on small-sample statistics theory, which finds out the optimal classification hyperplane by maximizing the geometric interval between the classification hyperplane and the data (25), and determines the constraint parameters of the model through the geometric interval. SVM method can map complex nonlinear problems to high-dimensional space and transform them into linear problems through kernel functions. The model algorithm can quickly train and learn to find the complex functional relationship between input and output. SVM is widely applied in classification of nonlinear, high-dimension, and small-size data samples. The optimization goal of SVM method is listed as follows.


(1) $$\min\frac{1}{2}\,{||\omega ||}^{2}+c\,\sum_{i=1}^{n}\xi_{i},\xi_{i}\geq 0$$

(2) $$s.t=\{\begin{array}{c} y_{i}\,\left(\omega \,x_{i}+b\right)\geq 1-\xi_{i}\\ c\geq 0 \end{array},i=1,2,3\,\ldots \,n$$

In Formula (1), *n* represents the number of calibration samples. In Formula (2), *x*_*i*_ is the support vector of calibration sample; *y*_*i*_ is the category of corresponding sample, whose value range is [–1, 1]. ω is the normal vector of hyperplane; *b* is offset vector. *c* is penalty factor. *ξ*_*i*_ is slack variable.

As two representative shallow learning methods, PLS-DA and SVM are both widely employed in Vis-NIR qualitative analysis. Both methods have their own characteristics of spectral data processing, PLS-DA is suitable for processing linear problems while SVM for nonlinear problems, but these two methods can neither select feature variables. When the data amount is large or the difference between spectral data is small, PLS-DA and SVM show poor classification performance and heavily rely on spectral data. Under such circumstances, a combination of spectral pretreatment methods and feature selection methods is necessary to obtain desirable classification effect.

#### Deep learning method

Convolutional Neural Network (CNN) is one of the widely studied deep learning algorithms with characteristics of local connection, weight sharing and down-sampling (26). The traditional CNN model is mainly composed of convolution layer, pooling layer and full connection layer. The convolutional layer extracts the features of the local sensing domain through the convolution kernel and non-linear transformation, and uses the activation function to filter the extracted features, thus effectively extracting the features of the input image while avoiding the problem of insufficient linear computing ability. Pooling layer, also known as sampling layer, down sampling the feature map according to rules, and reduces the dimension of the feature quantity after convolution in order to lessen the number of parameters and computation inside CNN and inhibit the network over-fitting at the same time. As the output layer, the full connection layer can not only connect the neurons of the upper and lower layers, but also output the weight score of each category through the activation function to obtain the final classification results. The traditional CNN model pays little attention to the extracted key features, so part of the noise information is retained and part of the key features are lost during feature transmission, which has a great influence on the model results (27). In view of the shortcomings of the traditional CNN model, the paper exerts more attention on the key features in the spectrum by using attention mechanism, so as to improve its ability of capturing key feature information and thus improve the recognition rate of the model for insect-affected Yali pears.

**FIGURE 4:**
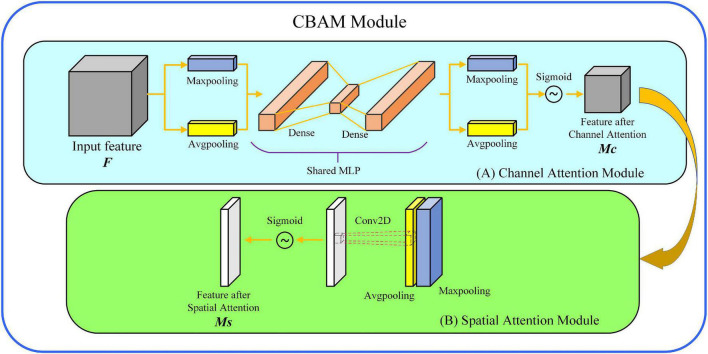
Convolutional block attention module (CBAM): **(A)** Channel attention module, **(B)** spatial attention module.

The structure of CBAM module is shown in Figure 4. Convolutional Block Attention Module-Convolutional Neural Networks (CBAM-CNN) adds the CBAM attention module to the original CNN model which makes the original CNN network have characteristic attention and improves the classification accuracy of the model. The CBAM module is an integrated module combining the Chanel Attention Module and the Spatial Attention Module, which can adaptively focus on the regions with obvious changes of feature points in the spectral matrix (28). Compared with a single attention module, the CBAM module can adaptively focus on “where are the important features” in the space relations and “which features are more important” in the channel relations, so that spectral features after a series of convolution can get more attention in CBAM module, and the corresponding weight information can be obtained in the full connection layer, so as to optimize the classification results.

### Research procedures

**FIGURE 5:**
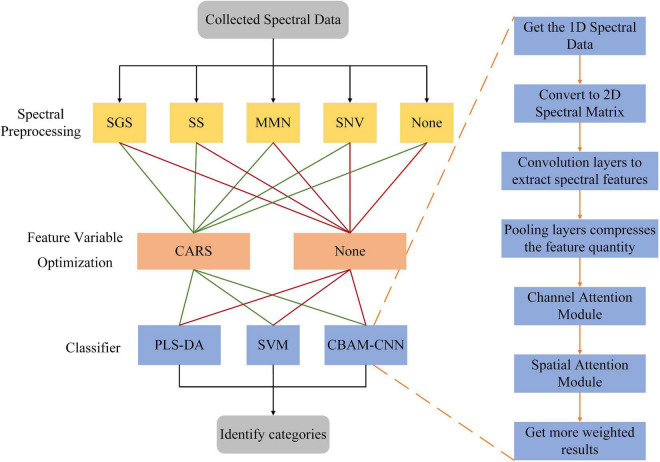
Research method flowchart.

For the collected spectral data, this paper has realized the identification of insect-affected pears in three steps. The flowchart of the research method is shown in Figure 5. The first step, the necessary spectral pretreatment is a key step to reduce spectral noise. This step in this paper is realized by the SGS, SS, MMN, and SNV methods. The second step, the optimization of feature variables is also an important step to improve the recognition rate. It has selected out more representative spectral bands in the entire spectrum. This step in this paper has been achieved by using the CARS method. In the third step, the choice of classifier also affect the recognition results. Three different classifiers are selected in this paper, including PLS-DA, SVM shallow learning models and CBAM-CNN deep learning model. For the CBAM-CNN deep learning model, its structural parameters are relatively complex than those of the shallow learning model. The CBAM-CNN model mainly consists of three parts, spectral data reading and conversion, feature extraction from convolution pooling and CBAM modules, and output model recognition results. Through three steps, the final Yali pear recognition category is obtained, and the optimal Yali pear recognition model is found.

### Evaluation indexes of models

In this paper, classification accuracy (Accuracy), classification accuracy of healthy pears (RH) and classification accuracy of insect-affected pears (RB) were used to comprehensively evaluate the prediction accuracy of the model. When the accuracy, RH, and RB values are closer to 100%, the classification performance is better. The evaluation indexes can be calculated according to the following Formulas (3–4).


(3) $$A\,c\,c\,u\,r\,a\,c\,y=\left(1-\frac{H_{e}+B_{e}}{H+B}\right)\times 100\%$$

(4) $$R\,H=\left(1-\frac{H_{e}}{H}\right)\times 100\%$$

(5) $$R\,B=\left(1-\frac{B_{e}}{B}\right)\times 100\%$$

Where, *H* is the total number of healthy pears; *B* is the total number of insect-affected pears; *H*_*e*_ is the number of healthy pears misclassified as insect-affected pears; *B*_*e*_ is the number of insect-affected pears misclassified as healthy pears.

## Results and analysis

### Visible-near-infrared spectral analysis of healthy pears and insect-affected pears

**FIGURE 6:**
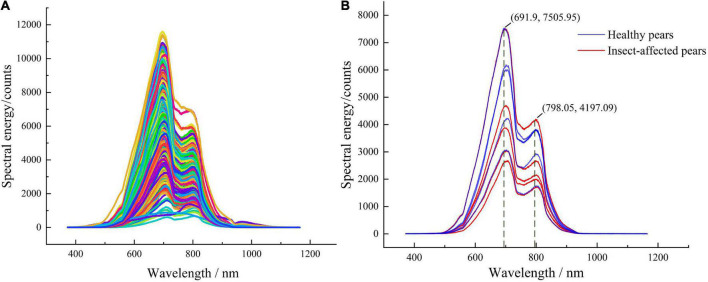
Vis-NIR spectra of healthy pears and insect-affected pears **(A)** original Vis-NIR spectra, **(B)** spectra of some samples.

The Vis-NIR spectra of Yali pears are shown in Figure 6A, in which each curve represents an individual Yali pears sample. It can be seen that the spectra of healthy pears and insect-affected pears are highly similar, and it is difficult to distinguish the spectral curves of the two categories. Five spectra were randomly selected from each category of sample spectra (as shown in Figure 6B). From the randomly selected 10 spectra of Yali pears, it can be seen that the spectra of healthy pears and insect-affected pears present obvious absorption peaks at the range of 691–798 nm, which is due to the information of color and pigment analysis (such as chlorophyll) of Yali pears in the spectral range of 400–750 nm, and the information of macro components (such as water and carbohydrate) of Yali pears in the spectral range of 750–2500 nm (29). From the spectral curves of healthy Yali pears and insect-affected pears, it can be seen that there is a phenomenon of spectral mixing between the two, and the clear threshold segmentation line cannot be found. Therefore, it is difficult to distinguish from the spectral figure whether Yali pears are damaged by insect-affected or not.

**FIGURE 7:**
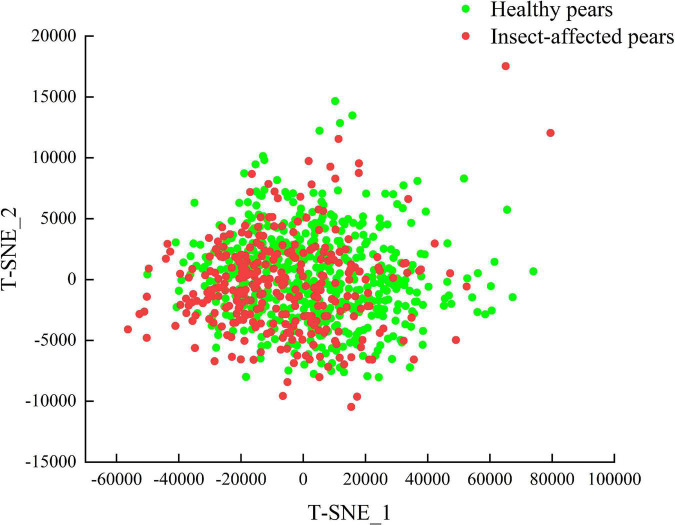
Feature visualization of healthy pears and insect-affected pears.

The spatial distribution of two kinds of pear samples in calibration set is analyzed by T-distributed Stochastic Neighbor Embedding (T-SNE) method. The T-SNE method is a nonlinear data-driven tool for dimension-reduction and visualization, which shows better performance in data visualization compared with other tools (30). The feature visualization of T-SNE method is shown in Figure 7. It can be seen from the figure that the spectral spatial distribution of healthy pears and insect-affected pears are intertwined, indicating that the T-SNE method cannot visually distinguish the spatial distribution of healthy pear samples and insect-affected pear samples. Hence, it is necessary to further explore the effectiveness of supervised pattern recognition in the identification of insect-affected Yali pears.

**FIGURE 8:**
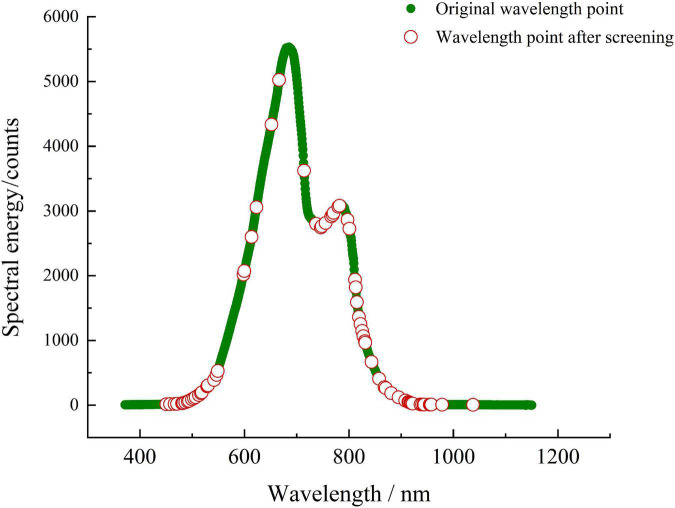
Distribution of feature spectral variables for Yali pears selected by CARS method.

In view of the large amount of spectral data of Yali pears and the redundancy and overlap of spectral information, the CARS algorithm is used to select feature variables to reduce the high collinearity between spectral variables. Through each adaptive weighted sampling, the variables with larger absolute weight of regression coefficients in PLS model are retained as new subsets and the variables with smaller weight are removed. In the algorithm implementation of the CARS method, the range of Monte Carlo randomness is 20–50, the sampling step is 1, and the sampling ratio is between 0.2 and 0.8. When the sampling times are 27 and the sampling rate is 0.8, the RMSECV is the minimum value of 0.33. After CARS screening, a total of 70 wavelength variables were selected, and the combination of wavelength variables selected at this time has the best effect. The distribution of feature spectral variables of Yali pears selected by CARS method is shown in Figure 8. It can be observed that wavelength points are mainly concentrated at the range of 450–550, 740–800, and 810–980 nm, which contain the main spectral information of Yali pears.

### Analysis on partial least squares discriminant analysis discrimination model of insect-affected Yali pears

The selection of the number of principal factors is the key to establish the model of Yali pears using the PLS-DA method. The leave-one-out cross validation is used to determine the optimal main factor number of the model, and the Accuracy and RMSECV of calibration set generated in the PLS model are calculated. The number of factors corresponding to the minimum RMSECV is selected as the optimal number of factors. The calibration results of different pretreatment methods and CARS modeling variable optimization methods combined with PLS-DA model are shown in Table 2.

**TABLE 2:** Calibration results of PLS-DA models based on different pretreatment methods and modeling variables.

Modeling variables	Pretreatment	Accuracy/%	RH/%	RB/%
All variables	None	80.73	88.98	66.89
	SGS	84.89	88.68	79.00
	MMN	76.43	85.47	62.33
	**SS**	**86.46**	**89.32**	**82.00**
	SNV	71.88	82.05	56.00
Selected variables	None	82.81	86.567	76.92
	SGS	85.42	88.46	80.67
	MMN	80.08	84.61	73.00
	**SS**	**87.37**	**90.39**	**82.67**
	SNV	73.18	82.27	59.00

It can be seen from Table 2 that before the CARS method feature variables are optimized, SS pretreatment method shows better classification results in the calibration set, with an accuracy of 86.46%. However, the classification results obtained by MMN and SNV pretreatment methods are not as good as the original spectral classification results, indicating that these two pretreatment methods are not suitable for the original spectral data. After the optimization of the CARS method feature variables, the number of features become less and the high collinearity among spectral bands is reduced, so the classification results of original data and processed spectra by pretreatment methods are both improved. The SS pretreatment method still shows good classification results in calibration set, with the accuracy of 87.37% and the number of selected principal factors of 15. In conclusion, the modeling effect of PLS-DA is better after being pretreated by the SS-CARS method.

### Analysis on support vector machine discrimination model of insect-affected Yali pears

The Grid Search (GS) is used to optimize the kernel function, *c* and *g* of the SVM model to achieve the optimal classification result. The calibration results of different pretreatment methods and the CARS method feature variable optimization methods combined with SVM model are shown in Table 3.

**TABLE 3:** Calibration results of SVM models based on different pretreatment methods and modeling variables.

Modeling variables	Pretreatment	Accuracy/%	RH/%	RB/%
All variables	None	75.00	75.601	73.568
	SGS	80.59	83.72	75.59
	MMN	83.59	84.76	81.52
	SS	84.51	84.42	84.67
	**SNV**	**87.89**	**86.69**	**90.27**
Selected variables	None	80.33	83.37	75.43
	SGS	81.38	83.51	77.74
	MMN	87.24	87.63	82.82
	SS	90.88	92.16	88.85
	**SNV**	**92.06**	**90.95**	**94.10**

It can be seen from Table 3 that before the optimization of the CARS method feature variables, the SNV pretreatment method shows good classification results in the calibration set, with an accuracy of 87.89%. The SGS, MMN, and SS pretreatment methods have improved the original spectral method, and the accuracy rate increases from 75% to more than 80%. After the optimization of the CARS method characteristic variables, the SS and SNV pretreatment methods show good classification results, with an accuracy of over 90%. The SNV pretreatment method presents the best classification results, with an accuracy of 92.06%, in this case, the selected kernel function is RBF, *c* is 50, and *g* is 3. In conclusion, the modeling effect of the SVM is better after the SNV-CARS pretreatment method.

### Analysis on convolutional block attention module-convolutional neural networks discrimination model of insect-affected Yali pears

**FIGURE 9:**
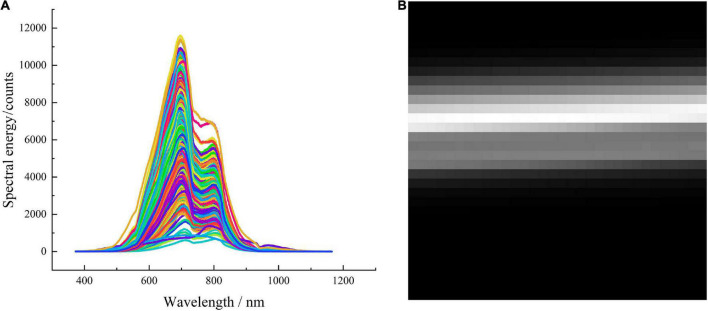
Original spectra of Yali pears and the transformed two-dimensional spectral image **(A)** original spectra, **(B)** two-dimensional spectral image.

Since the input data type of the CBAM-CNN model is two-dimensional image data while the original spectral data is one-dimensional data, so before the CBAM-CNN modeling process, it is necessary to transform one-dimensional data into two-dimensional image matrix and input the model in the form of image matrix. In the process of two-dimensional image matrix transformation, it is necessary to intercept the fixed length vector from front to back, stack the intercepted vector according to the line, and stack all the vectors in turn to form a two-dimensional image matrix. In this paper, 1,024 waveband points in the whole waveband are selected, and a row vector is formed by 32 waveband points in each row to evenly separate the whole waveband. Finally, the formed row vectors are stacked to create a 32 × 32 two-dimensional spectral matrix, and the visualization of the two-dimensional spectral matrix is shown in Figure 9.

**FIGURE 10:**
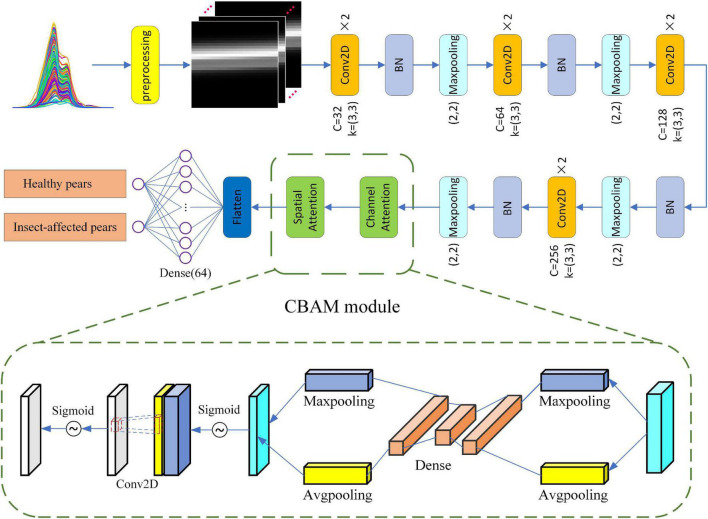
Structure diagram of CBAM-CNN model.

The structure of CBAM-CNN model is shown in Figure 10. In the construction of online Yali pear model through CBAM-CNN, convolutional layer, batch normalization (BN) layer, maxpooling layer, flattening layer and full connection layer are used. First, the network first carries out two-dimensional spectral matrix conversion, spectral pretreatment and feature variable optimization of spectral data. Second, the optimized two-dimensional spectral matrix is input into the model. The model first extracts the characteristics from the spectral matrix through the convolution layer, and then adds the maxpooling layer after each convolution layer to remove redundant information by down-sampling. Third, the convergence rate of the model training is accelerated by BN layer to prevent gradient explosion; the flattening layer compresses the output features from the pooling layer to compress the two-dimensional feature data into one-dimensional features. Last, the category with the largest weight score is output by *Sigmoid* activation function as the final model classification results.

In the network structure of the CBAM-CNN model, the BN layer is added to solve the problem of gradient disappearance and accelerate the convergence speed of the network, which has a certain regularization effect. Since there has some over-fitting of the network during training, L2 regularization term is added to limit weight information, and the Dropout layer is added to randomly shut down 20% of the neurons. In the setting of hyperparameters, *Relu* activation function and Adam optimizer are used to automatically reduce the learning rate. The initial learning rate is 0.001. The loss value doesn’t decrease every two times during training, the learning rate decays to 0.1 times of the previous time, with 50 batches and 300 epochs in total. The calibration results of different pretreatment methods and CARS feature variable optimization methods combined with CBAM-CNN model are shown in Table 4.

**TABLE 4:** Calibration results of CBAM-CNN models based on different pretreatment methods and modeling variables.

Modeling variables	Pretreatment	Accuracy/%	RH/%	RB/%
All variables	None	85.42	86.35	83.95
	**SGS**	**96.88**	**97.44**	**96.00**
	MMN	88.41	95.18	77.32
	SS	94.53	95.65	92.63
	SNV	90.62	92.97	87.68
Selected variables	None	86.85	89.73	82.13
	SGS	80.21	83.93	74.24
	**MMN**	**87.76**	**90.39**	**83.39**
	SS	83.33	93.97	65.51
	SNV	75.39	77.99	71.13

It can be seen from Table 4 that the SGS pretreatment method shows the best classification result in the calibration set without the optimization of CARS feature variables, with an accuracy of 96.88%. The MMN, SS, and SNV pretreatment methods all present better accuracy than the original spectral method. After the optimization of CARS feature variables, the MMN pretreatment method shows good classification results, with an accuracy of 87.76%; but the overall classification results are poorer than those before the selection of CARS feature variables. The reason behind worse classification results is that although the nonlinear problem between spectral data reduced after feature variables are selected by the CARS method, the number of spectral data features also reduced, leading to the insufficient feature quantity that can be learned by the deep learning model.

The validity of CBAM module is verified by constructing CNN model. During the construction of CNN model, the network structure, hyperparameters and the optimization scheme of spectral data of CNN model are consistent with those of the CBAM-CNN model. The test verifies that for the CNN model pretreated by SGS method, the calibration accuracy, RH and RB reach 95.18, 96.15, and 93.67%, respectively. Compared with the CBAM-CNN model, the accuracy decreases from 96.88 to 95.18%, indicating that the CBAM-CNN model has more attention to key feature points in spectral data than CNN model, thus improving the classification effect of the model on the insect-affected pears.

### Analysis on optimal model and discrimination mechanism of insect-affected Yali pears

The optimal results of PLS-DA model, SVM model and CBAM-CNN model are selected to analyze the specific classification of Yali pears in the validation set for each model, as shown in Table 5.

**TABLE 5:** Classification results of the validation set samples by PLS-DA, SVM, and CBAM-CNN models.

Model	Pretreatment	Sample status	*n*	*n*_*c*_	*n*_*w*_	Accuracy (%)	RH (%)	RB (%)	Prediction time(s)
PLS-DA	SS-CARS	Healthy	124	117	7	90.63	94.36	83.82	0.018
		Insect-affected	68	57	11				
SVM	SNV-CARS	Healthy	124	115	9	81.25	92.74	60.29	0.025
		Insect-affected	68	41	27				
**CBAM-CNN**	**SGS**	Healthy	124	119	5	**92.71**	**95.97**	**86.76**	**0.032**
		Insect-affected	68	59	9				

^a^*n*: Number of samples in the validation set.

^b^*n_c_*: Correct classification.

^c^*n_w_*: Wrong classification.

It can be seen that there is a small amount of imbalance in the spectral data of calibration set, resulting in more or less classification bias in the classification of each model, that is, the classification accuracy of the healthy pears (RH) is higher than that of the insect-affected pears (RB).

As classified by the PLS-DA model, the accuracy of validation set, RH, and RB are 90.63, 94.36, and 83.82%, respectively. A total of 18 Yali pears are misclassified, demonstrating that the classification results are average. The PLS is sensitive to the difference between groups, but the difference between spectral data groups of Yali pears in this experiment is large, thus leading to the misclassification. As classified by the SVM model, the accuracy of validation set, RH, and RB are 81.25, 92.74, and 60.29%, respectively. A total of 36 Yali pears are misclassified, demonstrating that the classification results are poor. Although the spectral data is pretreated, there is still overlapping of data points aliasing, which cannot be separated by a suitable hyperplane; besides, *c* and *g* obtained by the GS method are not the optimal parameters.

As classified by the CBAM-CNN model, the accuracy of validation set, RH, and RB are 92.71, 95.97, and 86.76%, respectively. A total of 14 Yali pears are misclassified, demonstrating that the classification results are the best. Due to the increase of attention module in the network, more attention is paid to the spectral characteristics in the spatial dimension and channel, thereby reducing the impact of unbalanced data. The misclassification of 14 pears results from the similarity of two types of original spectral data, and the difference between them fails to be amplified after the pretreatment by SGS. But in general, the CBAM-CNN model only pretreated by SGS shows better performance than other models.

The validation set accuracy of the traditional shallow PLS-DA learning model is 90.63%, and the prediction time of a single pear is 0.018 s, which is relatively short. The validation set accuracy of CBAM-CNN deep learning model is 92.71%, and the prediction time of a single pear is 0.032 s, which takes relatively long computation time. In the actual online sorting, the detection time of a single pear is mainly calculated by the integration time of the spectrometer and the prediction time of the sample spectrum of the model. In this case, the integration time is about 0.08 s and the prediction time is 0.032 s, so the total prediction time of a single pear is 0.112 s. In the production line, a single fruit cup conveyed six pears per second, and the average transmission time of a single fruit cup is 0.167 s. The verification results show that the total predicted time of a single pear is less than the transmission time of a fruit cup, and the time difference meets the requirements of online analysis.

### Interpretation of convolutional block attention module-convolutional neural networks deep learning model

In this paper, the T-SNE method is adopted to visualize the output features of CBAM-CNN model, so as to analyze the data clustering of Yali pear samples by the model. The Gradient-weighted Class Activation Mapping (Grad-CAM) is used to visualize the attention area of CBAM module, in a bid to locate the attention distribution in the spectral features.

**FIGURE 11:**
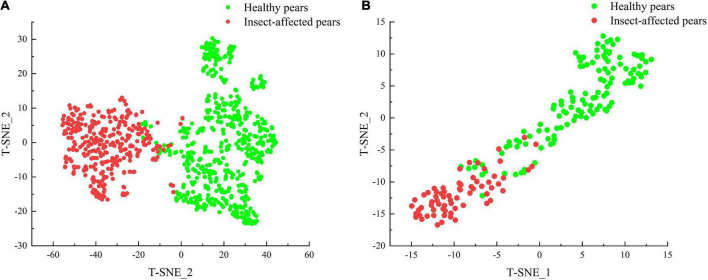
Visualization of output features by T-SNE: **(A)** Calibration set features distribution, **(B)** validation set features distribution.

The visualization of output features by T-SNE is shown in Figure 11. It can be observed that compared with the visualization of original spectral data features in Figure 7, the CBAM-CNN model has obvious classification effect for the two types of spectral data, as there is only a small amount of overlapping in the contact area rather than in the concentration area of the two types.

**FIGURE 12:**
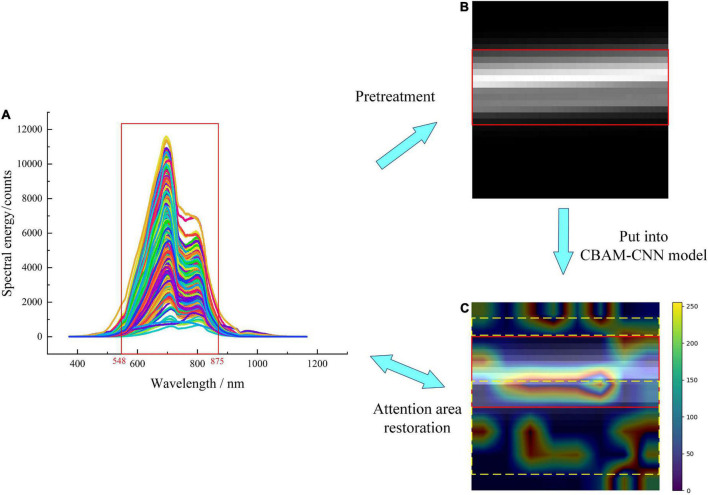
Visualization of Grad-CAM attention area: **(A)** Original spectra, **(B)** original spectral matrix image, **(C)** attention area spectral image.

The Grad-CAM method is used to generate class activation mappings for localizing the portion of spectral features displayed in the attention module. The feature heatmap is superimposed with the original image, which further shows the localization of some regions in the image by the network, and more intuitively explains the learning ability of the network (31). The color bar in the figure indicates the grading weight; as the color changes gradually from blue to red, and the corresponding weight value also increases. The larger the weight value, the stronger the ability of the model to learn features. The visualization of the attention area of Grad-CAM is shown in Figure 12. The highlight part in Figure 12B corresponds to the waveband at 548–875 nm in Figure 12A, which contains the entire wave peak of the spectrum region and is more representative.

The localization of the attention area of spectral matrix image by CBAM module is shown in Figure 12C. It can be seen that the attention modules can effectively pay attention to the wavebands corresponding to wave peaks tending to rise or fall in Figure 12A of the original spectral image (solid red line-box area), and the wavebands at 450–550, 740–800, and 810–980 nm optimally selected by CARS (yellow dotted line-box area). Besides, the highlight part and the optimally selected wavebands are given more red bands with high weight values, and targeted learning has been carried out.

Through the visualization analysis of the optimal model and feature, it can be seen that the classification of the deep learning model after spectral pretreatment on Yali pear pests is better than that of the shallow learning model after pretreatment and CARS feature variables optimization. The deep learning model itself can automatically extract features and learn, and use activate functions, attention mechanism and pooling layer, thus realizing the dual role of feature extraction and feature variable selection of spectral data. Therefore, the CBAM-CNN deep learning model shows better performance in discriminating insect-affected Yali pears than the PLS-DA and SVM shallow learning models.

## Conclusion

This paper proposes a non-destructive, rapid and online method to detect internal defects of Yali pears based on Vis-NIR, in order to rapidly find out the recessive insect-affected Yali pears during commercial sorting. Different pretreatment methods have been adopted in combination with CARS feature variables optimization to establish the PLS-DA and SVM shallow learning models and the CBAM-CNN deep learning model for online discrimination. The T-SNE and Grad-CAM are used to cluster the output characteristics of the model and visualize the attention area. The experimental results show that the recognition accuracy of PLS-DA and SVM shallow learning online discriminant model improved and is improved to more than 80% after spectral pretreatment and CARS feature variables optimization. The online discriminant model established based on spectra pretreated by SGS combined with CBAM-CNN deep learning method shows the best performance, the accuracy of calibration set and validation set is 96.88 and 92.71%, respectively, and the prediction time of single Yali pear is 0.032 s. Compared with shallow learning method, the deep learning method makes full use of the its autonomous feature extraction and learning ability, thus simplifying the modeling process and obtaining good feature clustering and attention areas of the models. The Vis-NIR model proposed in this paper meets the requirements of accuracy and time for online detection; Hence, it can be applied to detect insect-affected Yali pears during commercial sorting in the coming future.

## Data availability statement

The raw data supporting the conclusions of this article will be made available by the authors, without undue reservation.

## Author contributions

YH: theoretical methods, funding acquisition, manuscript writing, and writing—review and editing. CZ: experiments, software, method implementation, and writing and editing. XL: experimentation, supervision, and writing—review and editing. ZL: funding acquisition and writing—review. All authors contributed to this article and approved the submitted version.
